# Heterotrophy promotes the re-establishment of photosynthate translocation in a symbiotic coral after heat stress

**DOI:** 10.1038/srep38112

**Published:** 2016-12-05

**Authors:** Pascale Tremblay, Andrea Gori, Jean François Maguer, Mia Hoogenboom, Christine Ferrier-Pagès

**Affiliations:** 1 Centre Scientifique de Monaco, Monaco; 2 LEMAR - UMR 6539 UBO/CNRS/IRD, Institut Universitaire Européen de la Mer, Place Nicolas Copernic, Plouzané, France; 3 School of Marine and Tropical Biology, James Cook University, Townsville, Queensland, Australia

## Abstract

Symbiotic scleractinian corals are particularly affected by climate change stress and respond by bleaching (losing their symbiotic dinoflagellate partners). Recently, the energetic status of corals is emerging as a particularly important factor that determines the corals’ vulnerability to heat stress. However, detailed studies of coral energetic that trace the flow of carbon from symbionts to host are still sparse. The present study thus investigates the impact of heat stress on the nutritional interactions between dinoflagellates and coral *Stylophora pistillata* maintained under auto- and heterotrophy. First, we demonstrated that the percentage of autotrophic carbon retained in the symbionts was significantly higher during heat stress than under non-stressful conditions, in both fed and unfed colonies. This higher photosynthate retention in symbionts translated into lower rates of carbon translocation, which required the coral host to use tissue energy reserves to sustain its respiratory needs. As calcification rates were positively correlated to carbon translocation, a significant decrease in skeletal growth was observed during heat stress. This study also provides evidence that heterotrophic nutrient supply enhances the re-establishment of normal nutritional exchanges between the two symbiotic partners in the coral *S. pistillata*, but it did not mitigate the effects of temperature stress on coral calcification.

The earth is undergoing a period of rapid global warming, driven by increasing levels of atmospheric greenhouse gases^1^, which is occurring at unprecedented rates when compared with historical records^2^. Climate model predictions suggest severe loss of biodiversity because habitat specialists have limited capacity to keep up with climate warming through acclimatization, adaptation or via range shifts^3^. Many species may thus experience conditions that are outside of their physiological tolerance range^4^. Marine organisms that live in shallow habitats are particularly sensitive to the rapid rise in sea surface temperature that occurs during heat waves, i.e. abnormally hot weather^5^,^6^. Consequently, significant changes in pelagic productivity and composition have been observed during the last decade^6^,^7^ as well as mass mortality events of benthic species^8^,^9^,^10^. For instance, tissue necrosis or mortality was observed in 30 benthic species from several different phyla in the Mediterranean Sea following positive summertime thermal anomalies that occurred between 1998 and 2003^8^. Similarly, benthic communities in tropical coral reefs and atoll lagoons have shown significant declines in diversity and catastrophic losses in coral cover^10^,^11^,^12^,^13^ as well as local mortalities in a wide range of species such as echinoderms^14^ or intertidal barnacles^15^ due to heat waves and thermal anomalies.

Climate change is likely to have a profound impact on the distribution of marine species. Those characterized by mutualistic relationships, which allow organisms to excel in otherwise marginal habitats, will be particularly affected. The mutualistic symbiosis that corals form with autotrophic dinoflagellates of the genus *Symbiodinium* allows them to act as ecosystem engineers in nutrient poor tropical environments. This symbiosis, however, retains stability and function only under a particular set of stable environmental conditions and is sensitive to small increases in seawater temperatures. Heat stress disrupts the association between corals and dinoflagellates leading to a decrease in the concentration of symbionts within coral tissue^16^,^17^. This phenomenon, commonly referred to as coral bleaching, is predicted to increase in frequency and severity due to climate change^18^. For example, with the El Niño southern oscillation (ENSO), 95% of the corals were severely bleached or dead in the northern of the Great Barrier Reef in early 2016.

For reef-building corals, symbionts, through their photosynthetic activity and the translocation of more than 80% of photosynthates to the coral host^19^,^20^, are essential for the growth and survival of their host. Corals utilize these autotrophic nutrients mainly for their daily metabolic needs but also for lipid synthesis^21^,^22^, which can represent significant energy reserves that corals rely on during bleaching to support their metabolism^23^,^24^, although it is not always the case^25^. Nevertheless, heterotrophic feeding (plankton predation, dissolved and particulate organic matter consumption) is an alternative nutrient source for corals^26^,^27^ that has been proposed as a mechanism to help corals survive bleaching events^28^. However, ocean warming is causing a decrease in the nutrient enrichment of surface waters, a decline in zooplankton abundance^29^,^30^,^31^ and direct shifts in zooplankton composition^30^. All together, these changes in food abundance and symbiont concentration within coral tissue will impact the energetic capacities of corals and thereby affect corals’ resistance and resilience to environmental stressors.

Corals have developed several strategies to maintain energy acquisition during and after a bleaching event, which are not all well understood. First, they can alter the taxonomic composition of their endosymbiont communities toward a greater abundance of more thermally-resistant symbionts^32^, but this can have negative consequences for their growth^24^,^33^. Second, corals can increase their heterotrophic feeding capacity^28^,^34^,^35^, wherein energy and nutrients acquired through plankton feeding are either directly used by the host for its own needs^28^,^36^ or translocated to the symbionts to improve their growth and photosynthetic efficiency^37^,^38^. However, not all coral species are able to increase their heterotrophic capacity^28^,^34^,^39^, and the effectiveness of this strategy to promote bleaching-tolerance of corals in general is therefore uncertain. Third, corals can also increase metabolic efficiency and reduce waste excretion of nutrients by lowering their respiration rates and/or decreasing their rates of release of organic matter (i.e. mucus) into the surrounding environment^23^,^40^. However, evidence in the literature indicates that bleaching can either stimulate the loss of organic matter^39^,^41^,^42^ or its uptake^35^. Finally, corals can increase translocation of photosynthates by the remaining symbionts to sustain the host metabolism^43^ or translocate carbon to their symbionts to enhance their growth and photosynthesis, which in turn will increase carbon translocation^38^. This final mechanism of carbon exchange between the symbiotic partners is poorly understood. Detailed studies of coral energetic, that trace the flow of carbon from symbionts to coral host and vice versa under different environmental conditions, are thus required.

To address the relationship between the energetic status of corals and their resistance and reliance to a heat stress, we aimed to understand the carbon budget of colonies of *Stylophora pistillata* (Esper, 1797) maintained under control and heat stress conditions (25 °C and 31 °C respectively) as well as of colonies sampled after the heat stress. In addition, we assessed how the heterotrophic feeding of the coral modifies this carbon budget. Our working hypothesis was that carbon acquisition, exchange between the two partners of the symbiosis and retention within the symbiont and coral host will change with heat stress and/or heterotrophy, as these two factors impact the symbiont concentration as well as their rates of photosynthesis and respiration. In addition, we hypothesize that heat stress and heterotrophy will have opposite and long-term effects on the carbon budget. Heat stress should enhance carbon retention and utilization by the symbionts and, therefore, decrease carbon translocation to the host. On the contrary, heterotrophy should maintain symbiont concentration and photosynthesis during heat stress and thereby maintain the carbon acquisition and translocation compared to stressed and unfed corals. For these experiments, we chose the branching scleractinian coral *S. pistillata* (Pocilloporidae), because it is a key species of many reefs and is known to be a good heterotroph and will likely respond to both heat stress and heterotrophy. The experimental approach was to apply a heat stress on unfed and fed corals and study how temperature and feeding, independently or in combination, change the host-symbiont relationship in terms of nutrient acquisition and exchange between the two partners. Knowledge of the factors that determine whether bleaching is likely to result in coral mortality will provide a better understanding of how coral reefs, and the ecosystem services, they provide to human societies are likely to change in the future.

## Results

### Energy acquisition during heat stress and recovery

**Figure 1:**
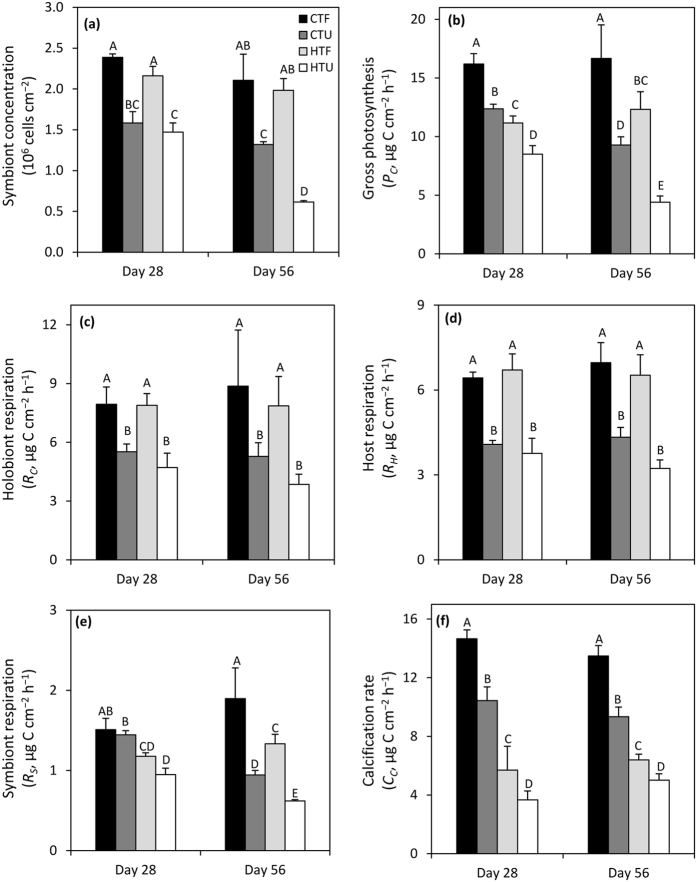
Effect of heat stress and heterotrophy on the main physiological parameters of *Stylophora pistillata*. (**a**) Symbiont concentration, (**b**) gross photosynthesis, *P*_*C*_, (**c**) holobiont respiration, *R*_*C*_, (**d**) host respiration, *R*_*H*_, (**e**) symbiont respiration, *R*_*S*_, and (**f**) calcification*, C*_*C*_, for fed and unfed nubbins over the course of heat stress (day 28) and recovering (day 56). Data are expressed as means ± standard error of the mean of *n* = 4 measurements. Bars with different letters (A to E) are significantly different (*p* < 0.05). CTF: control temperature and fed; CTU: control temperature and unfed; HTF: high temperature and fed; and HTU: high temperature and unfed. Day 28: samples in HTF and HTU were taken 28 days after the start of the heat stress at 31 °C; Day 56: samples in HTF and HTU tanks under the recovering process, taken 28 days after the end of the heat stress.

**Figure 2:**
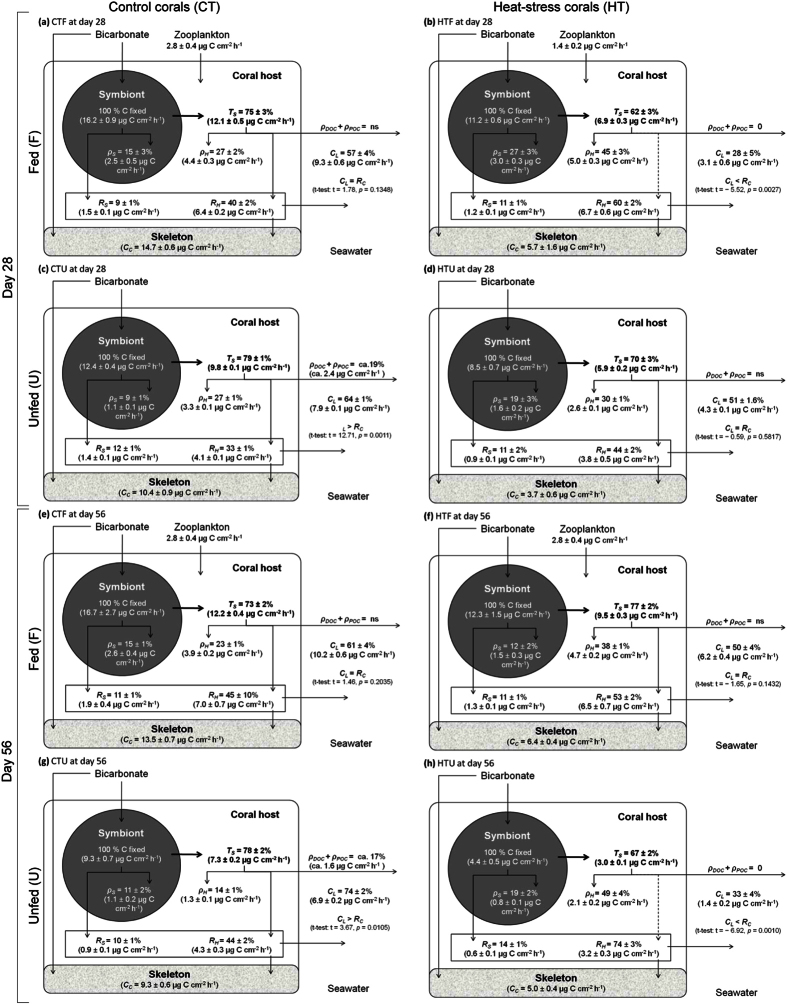
Mass-balanced results of photosynthate translocation and carbon budget of *Stylophora pistillata*. Results for nubbins at day 28 maintained fed under (**a**) control temperature at 25 °C (CTF) or (**b**) after the start of the heat stress at 31 °C (HTF), and unfed under (**c**) control temperature at 25 °C (CTU) or (**d**) after the start of the heat stress at 31 °C (HTU); at day 56 maintained fed under (**e**) control temperature at 25 °C (CTF) or (**f**) after the end of the heat stress at 31 °C (HTF), and unfed under (**g**) control temperature at 25 °C (CTU) or (**h**) after the end of the heat stress at 31 °C (HTU). Symbols are defined in the text. Data represent means and standard errors of means of *n* = 4 measurements. The significance of *ρ*_*DOC*_and *ρ*_*POC*_ was tested using a *t*-test on differences in the amount of carbon lost (*C*_*L*_ =* R*_*C*_ +* ρ*_*DOC*_ +* ρ*_*POC*_) and total holobiont respiration (*R*_*C*_). For fed corals, the zooplankton value was comes from Ferrier-Pagès *et al*.^34^ and Tremblay *et al*.^71^. Symbols are defined in the text, and a list of symbols and definitions is given in Table 5.

Significant changes in coral physiology occurred during heat stress (day 28) compared to the control condition (Figs 1 and 2). Although symbiont concentration remained equivalent to control corals (Fig. 1a and Table 1), heat-stressed fed and unfed nubbins (HSF and HSU) decreased their rates of gross photosynthesis by 31% and 46% respectively compared to control corals at day 28 (*P*_*C*_; Figs 1b and 2a–d, Table 1). As a consequence of lower carbon fixation by photosynthesis, the amount and percentage of carbon translocated was also lower in heat-stressed fed and unfed nubbins (*Ts*; Fig. 2a–d and Table 2, Fisher’s LSD test *p* < 0.05). The percentage of carbon incorporated by the symbionts (*ρ*_*S*_), however, significantly increased during the heat stress, both in fed (from 15% to 27%) and unfed corals (from 9% to 19%; Fig. 2a–d and Table 2, Fisher’s LSD test *p* < 0.001). In unfed corals, carbon incorporation rate in the coral host was also lower during the heat stress compared to control condition (*ρ*_*H*_; Fig. 2c,d and Table 2, Fisher’s LSD test *p* = 0.0356). Finally, CZAR and CHAR significantly decreased in fed corals, while CTAR significantly decreased in both fed and unfed corals (Table 3).

After the four weeks of recovery (day 56), several physiological parameters remained lower or even continued decreasing. The decline in physiological performance was most pronounced for the heat-stressed unfed corals (HTU) for which the following parameters were all significantly lower on day 56 compared with day 28: symbiont concentration (−58%; Fig. 1a and Table 1), gross photosynthesis (−48%; *P*_*C*_; Fig. 1b and Table 1), amount of photosynthate translocation (*Ts*; Fig. 2d,h and Table 2, Fisher’s LSD test *p* < 0.0001), CZAR and CTAR (Table 3). Interestingly, in control unfed corals (CTU), the gross photosynthesis (*P*_*C*_; Fig. 1b and Table 1), the amount of carbon translocated (*Ts*; Fig. 2c,g and Table 2, Fisher’s LSD test *p* < 0.0001) and incorporated in the host (*ρ*_*H*_; Fig. 2c,g and Table 2, Fisher’s LSD test *p* < 0.0001) as well as CZAR and CTAR (Table 3) were lower at day 56 than at day 28. For the heat-stressed and fed corals (HTF), the amount and percentage of carbon translocation increased at day 56 compared to day 28 (*Ts*; Fig. 2b,f and Table 2, Fisher’s LSD test *p* < 0.001) as well as CTAR and CHAR (Table 3).

### Energy expenditure during heat stress and recovery

During heat stress (day 28), respiration rates of the coral holobiont and host (*R*_*C*_ and *R*_*H*_), measured using the respirometry technique, were not affected compared to control conditions (Fig. 1c,d and Table 1). In contrast, symbiont respiration rates (*R*_*S*_), which represented less than 25% of the holobiont respiration, were significantly lower in heat-stressed versus control corals (Fig. 1e and Table 1). The amount and percentage of autotrophic carbon respired also decreased in heat-stressed corals (HTU and HTF) compared to control conditions (*C*_*L*_; Fig. 2a–d and Table 2, Fisher’s LSD test *p* < 0.05). After the recovery period (day 56), rates of symbiont respiration had declined further in unfed heat-stressed (HTU) corals (−53%; Fig. 1e and Table 1), and this was mirrored by a further decrease in the amount of carbon lost compared to day 28 (Fig. 2d,h and Table 2, Fisher’s LSD test *p* = 0.0023). Interestingly, symbiont respiration of the unfed control corals (CTU) also declined over this same time period (Fig. 1e and Table 1). Energy expenditure on calcification also differed over time. At day 28, heat-stressed fed and unfed nubbins had significantly decreased their rates of calcification (−61% and −65% respectively; Fig. 1f and Table 1), and this effect persisted throughout the recovery period (calcification rates were the same on day 56 compared to day 28).

### Comparison of the carbon budget of fed and unfed corals

Feeding significantly changed the carbon flux rates of control (Fig. 2a,c,e,g), heat-stressed (Fig. 2b,d), and recovering corals (Fig. 2f,h). For all treatments, feeding increased symbiont concentration (+47% minimum; Fig. 1a and Table 1) as well as rates of gross photosynthesis (+31% minimum; Fig. 1b and Table 1), holobiont respiration (+44% minimum; Fig. 1c and Table 1), host respiration (+58% minimum; Fig. 1d and Table 1) and calcification (+28% minimum; Fig. 1f and Table 1). The carbon budget was also significantly changed, with higher amounts of carbon translocated (*Ts*) and incorporated into the symbionts (*ρ*_*S*_) and host tissue (*ρ*_*H*_) of fed corals for all treatments (Fig. 2 and Table 2, Fisher’s LSD test *p* > 0.05).

**Figure 3:**
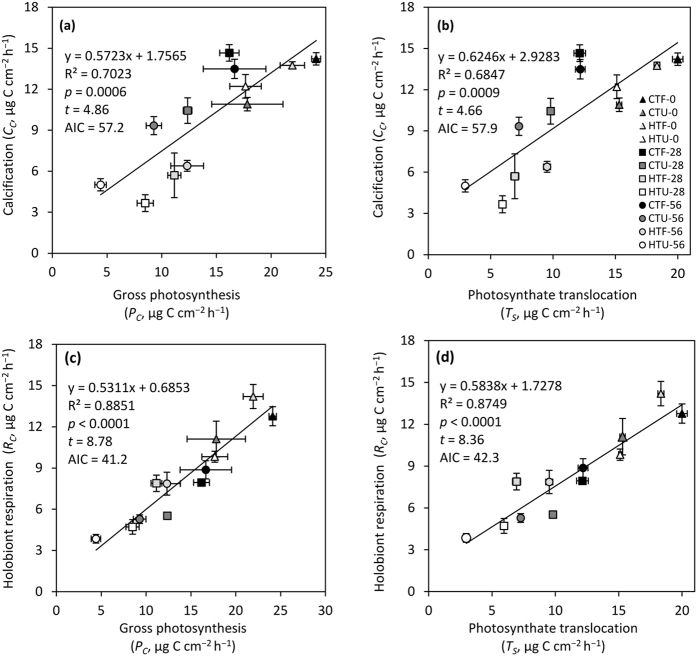
Relationship between autotrophic carbon and calcification or respiration rates of *Stylophora pistillata*. Relationship between calcification rates, *C*_*C*_, and (**a**) gross photosynthesis, *P*_*C*_, or (**b**) photosynthate translocation, *T*_*S*_, and between holobiont respiration, *R*_*C*_, and (**c**) gross photosynthesis, *P*_*C*_, or (**d**) photosynthate translocation, *T*_*S*_, for fed and unfed nubbins before (day 0), during (day 28) and after (day 56) the heat stress. Data are means ± standard errors of the mean of *n* = 4 measurements. Symbols are defined in the text, and a list of symbols and definitions is given in Table 5.

Feeding also promoted the recovery of corals from heat stress: when corals were fed, most components of the carbon budget (including, overall carbon fixation and translocation) had begun to returned to control levels (Fig. 2f), except for symbiont respiration and calcification rates which remained suppressed (Fig. 1e,f). Combined across all of the experimental treatments, our results demonstrate that calcification rates are strongly correlated with gross photosynthesis and carbon translocation (Fig. 3a,b). Hence, given that gross photosynthesis and carbon translocation were highly correlated (Pearson’s correlation r^2^ = 0.98), coral photosynthesis can be measured as a reliable proxy for coral growth. In addition, holobiont respiration was tightly correlated with photosynthesis and carbon translocation (Fig. 3c,d), suggesting that corals primarily respire autotrophic carbon.

## Discussion

A major challenge for ecology is to predict the effects of climate change on species performances and interactions^44^. The energetic status of corals, which is tightly coupled to the availability of inorganic and/or organic nutrients, is emerging as a particularly important factor that determines the corals’ vulnerability to stressors. The present study reveals the impact of heat stress on the nutritional interactions within a coral-dinoflagellate symbiosis. First, we demonstrate that, in the tropical reef-building coral *S. pistillata*, the fraction of photosynthetically assimilated inorganic carbon retained in the dinoflagellate cells was significantly higher during heat stress than under non-stressful conditions. This higher photosynthate retention in symbionts translated into lower rates of carbon translocation to the host, which required the coral to use tissue energy reserves to sustain its immediate respiratory needs. As calcification rates were positively correlated to carbon translocation in both fed and unfed colonies, a significant decrease in skeleton growth was observed during heat stress. This study also provides evidence that heterotrophic nutrient supply alleviates the negative effects of heat stress on coral calcification and enhances the re-establishment of normal nutritional exchanges between the two symbiotic partners.

Our results demonstrate that carbon exchange within symbioses can change substantially during heat stress, and that these changes can persist over relatively long time periods. We observed a sustained decrease in the amount of carbon translocated from symbiont to coral during and after the heat stress, mainly due to a significant decrease in the total amount of carbon fixed and in the percentage of carbon translocated to the host. This lower translocation was linked to a much higher percentage of carbon retained in symbionts during the stress. Although the contrary was also observed in corals^25^,^38^, a similar nutrient retention in symbionts was observed in lichens. In these later, while symbionts released up to 90% of their photosynthates to the fungal partner in optimal moisture conditions, they retained most of them during periods of water stress^45^. Different carbon translocation rates were also associated with differences in symbiont genotypes and physiology in the sea anemone *Aiptasia pallida*^46^. Higher retention of photosynthates by heat-stressed symbionts is likely linked to lipid synthesis by symbionts^22^ or to energy needs for repair of stress-induced damage to cells^38^, as suggested by the increased respiration rates of the symbionts. For instance, high temperatures disrupt the thylakoid membranes^47^ or the D1 protein of photosystem II^40^, which are essentials for efficient photochemical energy transduction. A re-allocation of energy to repair injured zones has often been observed in plant-myccorhizal associations^48^,^49^ or even in coral-endolith^50^ and coral-dinoflagellate^36^,^38^ associations. Nevertheless, despite retaining carbon for their own growth, symbionts of unfed *S. pistillata* were still unable to grow and divide rapidly enough to replace cells lost due to heat stress and, hence, symbiont numbers continued to decrease during the recovery period (day 56).

Contrary to unfed corals, normal nutritional exchanges between symbionts and the host in fed corals resumed as soon as the heat stress stopped. This restoration of photosynthate translocation (ca. 77% before and after the stress) was possible because symbiont growth and cell concentration were sustained by the heterotrophic feeding of the coral host. The importance of external food supply for the maintenance of symbiont concentration and photosynthetic activity has been observed several times^26^. Nevertheless, despite the additional energy and nutrients supplied to the symbiosis through feeding by the coral host, the symbionts still translocated less carbon to their host during the heat stress (carbon incorporation in symbionts increased from 15% to 27%), as also observed in the field^36^. This observation suggests that heterotrophic nutrients cannot entirely sustain symbiont needs during heat stress either because *S. pistillata* decreases its grazing rates under heat-stress^34^,^39^, or because nutrients are retained in the host tissue, or even because nutrients are not in a form that is suitable for translocation, although the contrary was suggested by Hughes *et al*.^38^. In a previous study, Tremblay *et al*.^51^ demonstrated that although heterotrophic nutrients can be rapidly translocated from the coral host to the symbionts, the majority of heterotrophic carbon remained in the host tissues. Collectively, these results suggest that carbon exchange is asymmetric between coral host and symbionts, and that bleaching can arise when nutrient allocation to symbionts is insufficient to afford the costs of repair mechanisms.

For fed colonies during heat stress, and for unfed colonies both during and after heat stress, total energy expenditure by the coral host exceeded the amount of translocated carbon, suggesting that the coral host had started respiring its tissue reserves and/or used heterotrophic nutrients (CTAR < 100%). In general, across many ecosystems and organisms, there is a tight coupling between rates of photosynthesis and respiration^52^,^53^,^54^, such that animal respiration decreases in response to declining photosynthetic carbon supply^52^, but it is not always the case^39^. Indeed, we observed an increase in the percentage of carbon respired during heat stress (from 33–45% to 53–74%; Fig. 2) despite a decrease in carbon fixation and translocation. Such an increase in energy expenditure on tissue maintenance during heat stress, so that enough adenosine triphosphate (ATP) is produced to sustain routine metabolism, has previously been observed^55^. The net effect of these adjustments in physiology was to maintain the same amount of carbon allocated to host tissue growth/reserves before and during the heat stress (4–5 μg C cm^−2^ h^−1^ in fed and 2–3 μg C cm^−2^ h^−1^ in unfed nubbins). It appears that these metabolic adjustments maintained the metabolic balance (i.e., energy homeostasis) but they are metabolically demanding processes^55^. The additional energy needed to cover these costs was partially obtained by heterotrophy in fed colonies, whereas energy supply and reserves were restricted in unfed colonies, and these colonies, therefore, experienced significant bleaching during the recovery period. These results are consistent with other studies that demonstrate that the amount of lipid stores in host tissue and the heterotrophic feeding capacity of a species underpin the resistance and/or resilience of corals to a single bleaching^23^,^24^,^56^,^57^. The carbon budgets of *S. pistillata* presented here actually show 30% higher respiration rates in fed compared to unfed colonies in all conditions and nearly constant respiration rates in unfed corals during the whole experiment. The higher rates of respiration in fed colonies may have contributed to a higher supply of metabolic CO_2_ to the symbionts, thereby maintaining a higher symbiotic stability^58^,^59^.

Metabolic re-adjustments came at the expense of calcification for both fed and unfed colonies during heat stress, highlighting that biomineralization is an energetically costly process^60^. Calcification was not directly related to respiration rates, but rather was associated with photosynthetic and carbon translocation rates. In addition, calcification rate was related to heterotrophic feeding^61^, with rates 1.3–1.6 times higher in fed compared to unfed nubbins. Heterotrophy could affect calcification directly through the supply of organic molecules or energy necessary to the building of the organic matrix^62^, or it might affect calcification indirectly by enhancing photosynthesis. Nevertheless, when photosynthesis was reduced in both fed and unfed corals at high temperature, calcification was also reduced by 61–65% compared to control temperature at day 28, and by 46–53% at day 56. These results suggest that heterotrophic feeding does not alter the sensitivity of corals to heat stress, as previously observed in corals under acidification stress^63^,^64^,^65^,^66^. Our observation is also in contrast to other studies demonstrating that supply of inorganic nutrients can reduce calcification sensitivity to both heat stress and to ocean acidification^66^,^67^,^68^,^69^. The most likely explanation for these contrasting results is that inorganic nutrients are primarily taken up by symbionts, whereas organic nutrients (feeding) are acquired by the host and a small fraction is subsequently shared with the symbionts. As calcification is related to photosynthesis, and photosynthesis is enhanced by inorganic nutrient supply, provision of inorganic nutrients can prevent decreases in calcification during heat stress. Nevertheless, fed corals at day 56 had the same rates of photosynthesis and carbon translocation as unfed corals in control conditions, but 30% lower calcification rates. We hypothesize that the difference in calcification observed is linked to the quality of the photosynthates produced. Stress-induced changes in exopolysaccharide composition and production have been observed in cyanobacteria^70^, and the same could potentially occur in corals. A better knowledge of the effect of photosynthate quality on calcification rates will provide new insight into the effect of climate change on coral calcification.

This work has applied multiple tools (physiological and isotopic labeling approaches) to the study of host-symbiont interactions and has revealed new data to illustrate how heat stress and heterotrophy interact, with implications for reef coral ecology in the midst of global change. By combining pulse-chase stable-isotopic labeling (^13^C) with physiological measurements, we have thus quantified the acquisition and allocation of carbon and nutrients within a coral-dinoflagellate symbiosis during a heat stress event. These results demonstrate that an elevation in seawater temperature induces a shift in carbon allocation toward retention of carbon by the symbionts and, consequently, decreased the amount of carbon translocated to the coral host. Provision of particulate matter as a heterotrophic food source for the coral host contributed to restoring the normal nutritional relationships between host and symbionts, but it did not mitigate the effects of temperature stress on coral calcification and growth rates. More broadly these results suggest that coral calcification, and the net accretion of coral reefs, is likely to be severely impacted as heat stress events intensifies in the future.

## Materials and Methods

### Coral samples

A total of 208 nubbins were prepared by cutting the apical branches of four large colonies (52 nubbins per colony) of the scleractinian coral *Stylophora pistillata* originating from the Red Sea. Nubbins were then distributed in eight 20 l tanks (*n* = 26 per tank, 13 nubbins per colony per treatment) and maintained for four weeks until tissue entirely covered the skeleton. During this period, all nubbins were fed twice a week with *Artemia salina* nauplii (2,000 nauplii per tank per feeding event^71^). They were maintained under an irradiance of 250 μmol photons m^−2^ s^−1^ (12 h light: 12 h dark cycle) in a flow through system supplied with freshly pumped seawater (renewal rate of 10 l h^−1^). The total daily light integral received by our corals in these conditions (10.8 mol photons m^−2^ d^−1^) is equivalent to the one received on reefs at 3 m depth^72^. A typical sunny day generates a total of 14.4 mol photons m^−2^ d^−1^ at 3 m depth, while a day with periodic cloud cover generated 11.9 mol photons m^−2^ d^−1^ and a cloudy day 6.1 mol photons m^−2^ d^−1^. Water temperature was adjusted to 25.0 ± 0.5 °C using heaters connected to electronic controllers. Seawater contained low levels of nitrate (<2 μM) and phosphates (<0.2 μM). Before the start of the experiment, feeding was ceased in four of the eight tanks, and these nubbins were maintained unfed for five weeks. Nubbins in the remaining four tanks were fed three times a week with *A. salina* nauplii over the same time period (2,000 nauplii per nubbins per feeding event^71^).

### Experimental design

After the preparation of fed and unfed nubbins, a set of initial measurements, described below, was performed in all tanks (called day 0, see Supplementary Information). Then, four different treatments (with two tanks per treatment) were established using a factorial design: nubbins were either kept unfed or fed at a control temperature of 25.0 ± 0.5 °C or at an elevated temperature of 31.0 ± 1.0 °C. Temperature was increased over 10 days, which corresponds to the temperature variations occurring in some reef flats or during low tide conditions^73^,^74^,^75^. Hereafter, these four treatments will be referred to as: CTF (control temperature at 25 °C and fed), CTU (control temperature at 25 °C and unfed), HTF (high temperature at 31 °C and fed), and HTU (high temperature at 31 °C and unfed). In the HTF and HTU tanks, heat stress was maintained for four weeks (until a decrease in the photosynthetic efficiency of the corals under heat stress was monitored), after which a set of measurements were performed in all tanks (called day 28). Temperature was then decreased over one week back to 25.0 ± 0.5 °C, and tanks were maintained for three additional weeks under these recovery conditions (same length as for the stress). A set of final measurements was performed in all tanks at the end of the fourth week of recovery (called day 56).

### Rates of calcification, respiration and photosynthesis

Calcification rates were determined at each sampling time (day 0, day 28 and day 56) for the same four nubbins per treatment (two nubbins per tank from four colonies), using the buoyant weight technique^76^. Carbon allocation to calcification (*C*_*C*_) was calculated according to the formula: *C*_*C*_ = *M*_*Sk*_ × 12/100, where *M*_*Sk*_ is the μg CaCO_3_ produced and 12/100 is the ratio of molecular masses of C (12) and CaCO_3_ (100).

Rates of respiration (*R*) and net photosynthesis (*P*_*n*_) were measured using four nubbins per treatment (two nubbins per tank from four colonies) at each sampling time. Measurements were performed using temperature–controlled chambers coupled with optodes (OXY-4 micro, PreSens, Germany) and filled with ~50 ml of 0.45 μm-filtered seawater (FSW). Optodes were calibrated before each measurement using nitrogen gas (N_2_) and air saturated water as 0 and 100% oxygen saturation values respectively. Seawater temperature in the chambers was maintained at 25.0 or 31.0 ± 0.5 °C, and water was continuously stirred using magnetic stirrers. For each colony, *P*_*n*_ was measured at the treatment irradiance of 250 μmol photons m^−2^ s^−1^, and *R* was also measured during 20 to 30 min. Rates of gross photosynthesis (*P*_*g*_) were calculated by adding *R* to *P*_*n*_. Samples were then frozen for later determinations of symbiont concentration using an inverted microscope (Leica, Wetzlar, Germany) and the Histolab 5.2.3 image analysis software (Microvision, Every, France), according to Rodolfo-Metalpa *et al*.^77^.

To apportion holobiont respiration into symbiont and host components, the respiration rates of freshly isolated symbionts (*R*_*S*_) were determined for four nubbins per treatment (two nubbins per tank from four colonies) at each sampling time. Symbionts were extracted in FSW using an air-brush, homogenised and then centrifuged at 850 g for 10 min to pellet the symbionts. The supernatant was discarded, and the symbionts were re-suspended in FSW. Respiration rates and symbiont concentration were measured as described above. The host respiration (*R*_*H*_) was obtained by subtracting *R*_*S*_ from *R*_*C*_.

The autotrophically-fixed (*P*_*C*_) and respired (*R*_*C*_) carbon were calculated by converting oxygen fluxes to carbon equivalents using the molar weights, as *P*_*C*_ = *P*_*g*_ × 12/*PQ* and *R*_*C*_ = *R* × 12 × *RQ*^78^, where *PQ* and *RQ* are photosynthetic and respiratory quotients equal to 1.1 mol O_2_:mol C and 0.8 mol C:mol O_2_, respectively^19^.

### H^13^CO_3_ labeling experiments

H^13^CO_3_ labeling experiments were performed according to Tremblay *et al*.^20^,^71^. For each treatment and sampling time, four nubbins (two per tank from four colonies) were incubated in individual beakers containing 200 ml of FSW enriched with a final concentration of 0.6 mM NaH^13^CO_3_ (98 atom %^13^C, #372382, Sigma-Aldrich, St-Louis, MO, USA), giving a final 23.5% ^13^C enrichment of the incubation medium. After a 5 h incubation period in the light (pulse), nubbins were transferred to new beakers filled with 200 ml of non-enriched FSW (chase). The medium was continuously stirred and maintained at 25.0 or 31.0 ± 0.5 °C using a water bath. Nubbins were removed from the incubation medium after a 48 h chase and immediately frozen at −20 °C. Four control nubbins per treatment and sampling time (two per tank from four colonies) were incubated in parallel in 200 ml non-enriched FSW. All nubbins were treated according to Tremblay *et al*.^20^,^71^. Briefly, tissue was detached from the skeleton using an air-brush in FSW. The slurry was homogenised using a potter tissue grinder and centrifuged to separate coral host and symbionts. Samples were flash-frozen in liquid nitrogen and freeze-dried until analysis. The atom percent of ^13^C and the carbon content of the coral host and symbionts were determined with a mass spectrometer (Delta Plus, Thermofisher Scientific, Bremen, Germany) coupled via a type III interface with a C/N analyzer (Flash EA, Thermofisher Scientific).

### Autotrophic and heterotrophic carbon budget calculations

The equations used to calculate the autotrophic carbon budget are fully described in Tremblay *et al*.^20^,^71^ and summarized in Table 4. These equations allow the calculations of the amount and percentage of fixed carbon lost (*C*_*L*_), which corresponds to the carbon lost as respiration by the symbiotic association (*R*_*C*_) as well as the carbon released as dissolved and particulate organic carbon (*ρ*_*DOC*_and *ρ*_*POC*_ respectively), the amount and percentage of carbon translocation from the symbionts to the host (*T*_*S*_), the carbon incorporation rates in the symbionts (*ρ*_*S*_) or coral host (*ρ*_*H*_), and the percentage of fixed carbon remaining (*C*_*R*_) in the symbionts or coral host. See Table 5 for a list of symbols and their definitions. All data were normalized to the skeletal surface area using the wax technique^79^.

The percentages of autotrophic carbon contributing to the total respiration of the holobiont (CZAR) have been assessed in each treatment^19^,^28^. CZAR was calculated taking into account the total amount of carbon fixed by the photosynthesis per hour (*P*_*C*_) during 12 h of photosynthesis divided by the holobiont respiration (*R*_*C*_) during 24 h (Table 4). The percentages of autotrophic and heterotrophic carbon contributing to the respiration of the host (CTAR and CHAR respectively) have also been assessed. CTAR was calculated taking into account the total amount of carbon translocated to the host per hour (*T*_*S*_) during 12 h of photosynthesis divided by the host respiration (*R*_*H*_) during 24 h (Table 4). CHAR^28^ was calculated assuming a grazing rate (*H*_*C*_) of 2.8 μg C cm^−2^ h^−1^ for corals maintained at 25 °C and 1.4 μg C cm^−2^ h^−1^ for those maintained at 31 °C^34^,^71^ during 24 h divided by the host respiration (*R*_*H*_) during 24 h (Table 4).

### Statistical analysis

All parameters were expressed as average value ± standard error of the mean (s.e.m.). Data were checked for normality using a Kolmogorov-Smirnov’s test with Lilliefors correction and for variance homogeneity using Levene’s test. Data were transformed with a natural logarithm transformation when required (i.e. symbiont concentration). The effect of treatment on the different parameters was tested using three-way analyses of variance (ANOVA), in which the effect temperature was assessed using a comparison between control (25 °C) and heat-stressed (31 °C) corals. The factors of interest were time after the start and the end of the heat stress (two levels: heat-stress at day 28 and recovering at day 56), temperature (two levels: control at 25 °C, CT, and high at 31 °C, HT), and feeding regimes (two levels: fed and unfed). When there were significant differences, the ANOVAs were followed by *a posteriori* test (Fisher’s LSD test). The tank effect nested within temperature and feeding was not significant for any of the parameters, it was therefore excluded from analyses. The two tanks per temperature and feeding were pooled, according to the procedure described in Underwood^80^, to check that variation among experimental units was zero, and that pooling was appropriate and did not change the conclusion of the analysis. In order to test whether *ρ*_*DOC*_ +* ρ*_*POC*_ was significantly different than zero we used a *t*-test to compare the amount of carbon lost (*C*_*L*_ =* R*_*C*_ +* ρ*_*DOC*_ +* ρ*_*POC*_) to total holobiont respiration (*R*_*C*_), with degrees of freedom calculated as d.f. = *nC*_*L*_ +* nR*_*C*_ − 2. If the *t*-value of this test was positive and *p*-value < 0.05 then *C*_*L*_ > *R*_*C*_ and *ρ*_*DOC*_ +* ρ*_*POC*_ > 0^20^. Linear regressions, using standard least-squares techniques, were used to estimate the relationship between the mean of *P*_*C*_ or *T*_*S*_ and the mean of *C*_*C*_ or *R*_*C*_. Differences between factors were considered significant for *p*-values < 0.05. Statistical analyses were performed using Systat 13 software (Systat Software, Chicago, IL, USA).

## Additional Information

**How to cite this article**: Tremblay, P. *et al*. Heterotrophy promotes the re-establishment of photosynthate translocation in a symbiotic coral after heat stress. *Sci. Rep.* **6**, 38112; doi: 10.1038/srep38112 (2016).

**Publisher's note:** Springer Nature remains neutral with regard to jurisdictional claims in published maps and institutional affiliations.

## Supplementary Material

Supplementary Information

## Figures and Tables

**Table 1:** Results of the three-way analyses of variance (ANOVA) testing the effect of time, temperature and feeding on physiological parameters in *Stylophora pistillata*.

Factor	d.f.	*p*	*F* value
*Symbiont concentration*			
Time	1	**0.0001**	20.21
Temperature	1	**0.0028**	10.65
Feeding	1	**<0.0001**	66.42
Time × Temperature	1	0.0508	4.15
Time × Feeding	1	**0.0155**	6.61
Temperature × Feeding	1	**0.0189**	6.18
Time × Temperature × Feeding	1	**0.0120**	7.19
Error	29	—	—
*Photosynthesis (P_C_)*			
Time	1	0.1731	2.00
Temperature	1	**0.0002**	21.53
Feeding	1	**<0.0001**	31.14
Time × Temperature	1	0.9391	0.01
Time × Feeding	1	**0.0357**	5.11
Temperature × Feeding	1	0.8769	0.02
Time × Temperature × Feeding	1	0.6710	0.19
Error	19	—	—
*Holobiont respiration (R_C_)*			
Time	1	0.9102	0.01
Temperature	1	0.0812	3.39
Feeding	1	**<0.0001**	54.28
Time × Temperature	1	0.3910	0.77
Time × Feeding	1	0.2770	1.25
Temperature × Feeding	1	0.5174	0.44
Time × Temperature × Feeding	1	0.8543	0.03
Error	19	—	—
*Host respiration (R_H_)*			
Time	1	0.9102	0.01
Temperature	1	0.0812	3.39
Feeding	1	<**0.0001**	54.28
Time × Temperature	1	0.3910	0.77
Time × Feeding	1	0.2770	1.25
Temperature × Feeding	1	0.5174	0.44
Time × Temperature × Feeding	1	0.8543	0.03
Error	19	—	—
*Symbiont respiration (R_S_)*			
Time	1	0.5201	0.43
Temperature	**1**	**0.0008**	15.78
Feeding	1	**0.0002**	20.54
Time × Temperature	1	0.8918	0.02
Time × Feeding	1	**0.0050**	10.08
Temperature × Feeding	1	0.8671	0.03
Time × Temperature × Feeding	1	0.3670	0.85
Error	19	—	—
*Calcification rate (C_C_)*			
Time	1	0.9251	0.01
Temperature	1	<**0.0001**	120.16
Feeding	1	<**0.0001**	22.65
Time × Temperature	1	0.0914	3.02
Time × Feeding	1	0.7713	0.09
Temperature × Feeding	1	0.0538	3.99
Time × Temperature × Feeding	1	0.8156	0.06
Error	34	—	—

Symbiont concentrations were ln-transformed prior to analysis. *n* = 4 replicates; significant *p*-values are in bold.

**Table 2:** Results of the three-way analyses of variance (ANOVA) testing the effect of time, temperature and feeding on autotrophic carbon parameters (carbon lost and translocation as well as incorporation rates and carbon fixed remaining in symbionts and coral host), after 48 hours in *Stylophora pistillata*.

Factor	d.f.	Amount (μg C cm^−2^ h^−1^)		Percentage (%)	
		*p*	*F* value	*p*	*F* value
*Carbon lost (C_L_)*					
Time	1	0.9562	<0.01	0.0972	3.00
Temperature	1	<**0.0001**	222.09	<**0.0001**	85.16
Feeding	1	<**0.0001**	39.96	**0.0223**	6.04
Time × Temperature	1	0.8313	0.04	0.3323	0.98
Time × Feeding	1	**0.0001**	37.97	**0.0029**	11.24
Temperature × Feeding	1	0.3738	0.82	0.1919	1.82
Time × Temperature × Feeding	1	**0.0053**	9.59	**0.0002**	20.59
Error	22	—	—	—	—
*Carbon translocation (T_S_)*					
Time	1	**0.0027**	11.47	0.1861	1.86
Temperature	1	<**0.0001**	351.23	**0.0003**	18.49
Feeding	1	<**0.0228**	5.99	**0.0378**	4.88
Time × Temperature	1	<**0.0001**	298.77	0.3076	1.09
Time × Feeding	1	<**0.0001**	90.81	**0.0209**	6.19
Temperature × Feeding	1	0.7312	0.12	0.1009	2.99
Time × Temperature × Feeding	1	**0.0021**	12.11	**0.0118**	7.54
Error	22	—	—	—	—
*Incorporation rate (ρ_H_) and fixed carbon remaining (C_R_) in coral host*					
Time	1	<**0.0001**	27.69	0.4498	0.59
Temperature	1	**0.0252**	5.77	<**0.0001**	129.86
Feeding	1	<**0.0001**	194.06	**0.0432**	4.60
Time × Temperature	1	**0.0104**	7.86	**0.0002**	20.33
Time × Feeding	1	**0.0198**	6.31	**0.0112**	7.66
Temperature × Feeding	1	**0.0331**	5.17	0.4377	0.62
Time × Temperature × Feeding	1	**0.0319**	5.25	<**0.0001**	31.07
Error	22	—	—	—	—
*Incorporation rate (ρ_S_) and fixed carbon remaining (C_R_) in symbionts*					
Time	1	**0.0115**	7.61	**0.0066**	3.73
Temperature	1	0.7137	0.14	**0.0011**	13.94
Feeding	1	<**0.0001**	34.46	0.0865	3.22
Time × Temperature	1	**0.0130**	7.31	**0.0183**	6.50
Time × Feeding	1	0.4376	0.63	**0.0167**	6.71
Temperature × Feeding	1	0.3383	0.96	0.1977	1.76
Time × Temperature × Feeding	1	0.3053	1.10	0.0765	3.47
Error	22	—	—	—	—

Significant values are in bold.

**Table 3:** Percentages of autotrophic carbon contributing to the total respiration of the holobiont (CZAR) as well as percentages of autotrophic and heterotrophic carbon contributing to the respiration of the host (CTAR and CHAR respectively), for fed and unfed nubbins of *Stylophora pistillata* over the course of heat-stress (day 28) and recovering (day 56).

	CZAR (%)	CTAR (%)	CHAR (%)	CTAR + CHAR (%)
	mean ± s.e.m.^(Fisher)^	mean ± s.e.m.^(Fisher)^	mean ± s.e.m.^(Fisher)^	mean ± s.e.m.^(Fisher)^
Day 28				
CTF	102.1 ± 6.7^(AB)^	94.3 ± 3.1^(AB)^	43.5 ± 1.4^(A)^	137.8 ± 4.5^(A)^
CTU	112.0 ± 1.7^(A)^	120.3 ± 4.3^(B)^		120.3 ± 4.3^(AB)^
HTF	71.0 ± 1.9^(D)^	52.1 ± 4.1^(C)^	21.1 ± 1.7^(B)^	73.2 ± 5.8^(CD)^
HTU	90.8 ± 2.6^(ABC)^	83.0 ± 11.0^(D)^		83.0 ± 11.0^(C)^
Day 56				
CTF	93.6 ± 13.2^(ABC)^	89.5 ± 10.0^(AD)^	41.0 ± 4.6^(A)^	130.5 ± 14.6^(A)^
CTU	87.8 ± 1.7^(BC)^	85.3 ± 6.6^(D)^		85.3 ± 6.6^(BC)^
HTF	78.1 ± 2.5^(CD)^	76.2 ± 7.9^(AD)^	44.8 ± 4.7^(A)^	121.0 ± 12.6^(A)^
HTU	56.9 ± 2.3^(E)^	47.5 ± 3.8^(C)^		47.5 ± 3.8^(D)^

Data are expressed as means ± standard error of the mean of *n* = 4 measurements. Numbers with different letters in parentheses (A to E) are significantly different (*p* < 0.05). Symbols are defined in the text, and a list of symbols and definitions is given in Table 5.

**Table 4:** Equations used to calculate the autotrophic and heterotrophic carbon budget.

Parameters		Equations	References
ρ*	=	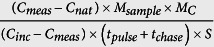	Tremblay *et al*.^20^,^71^
*C*_*inc*_	=	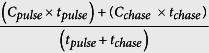	Tremblay *et al*.^20^
*P*_*C(theoretical)*_	=	*R*_*H*_ + *R*_*S*_ + *ρ*_*S*_ + *ρ*_*H*_ + *ρ*_*POC*_ + *ρ*_*DOC*_	Tremblay *et al*.^20^,^71^
*C*_L_*	=	*R*_*C*_ + *ρ*_*POC*_ + *ρ*_*DOC*_ = *P*_*C*_ − *ρ*_*S*_ − *ρ*_*H*_	Tremblay *et al*.^20^,^71^
*T*_S_*	=	*P*_*C*_ − *ρ*_*S*_ − *R*_*S*_	Tremblay *et al*.^20^,^71^
*ρ*_*POC*_ + *ρ*_*DOC*_	=	*C*_*L*_ − *R*_*C*_ = *(P*_*C*_ − *ρ*_*S*_ *−ρ*_*H*_) − *R*_*C*_	Tremblay *et al*.^20^,^71^
CZAR	=	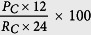	Muscatine *et al*.^19^ Grottoli *et al*.^28^
CTAR	=	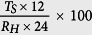	This paper
CHAR	=	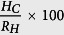	Grottoli *et al*.^28^

^*^The percentage of fixed carbon remaining (*C*_*R*_) in symbionts or coral host as well as the percentage of *C*_*L*_ and *T*_*S*_ was obtained by dividing *ρ* or *C*_*L*_ or *T*_*S*_ by *P*_*C*_, and multiplying by 100.

**Table 5:** List of symbols, definition and units.

Symbol	Definition (unit)
C	Carbon
*C*_*C*_	Amount of carbon used by calcification (μg C cm^−2^ h^−1^)
CHAR	Percentage of heterotrophic carbon contributing to the respiration of the host (%)
*C*_*chase*_	Atom percent of ^13^C in the non-enriched incubation medium (equal to 1.1%)
*C*_*inc*_	Atom percent of ^13^C in the enriched incubation medium (%)
*C*_*L*_	Autotrophic carbon lost (μg C cm^−2^ h^−1^ or %)
*C*_*meas*_	Atom percent of ^13^C measured in the enriched samples (%)
*C*_*nat*_	Natural atom percent of ^13^C in non-enriched nubbins (%)
*C*_*pulse*_	Atom percent of ^13^C in the enriched incubation medium (equal to 23.5%)
*C*_*R*_	Percentage of autotrophic carbon remaining in symbionts or host tissue (%)
CTF	Control temperature and fed corals
CTU	Control temperature and unfed corals
CTAR	Percentage of autotrophic carbon contributing to the respiration of the host (%)
CZAR	Percentage of autotrophic carbon contributing to the total respiration of the holobiont (%)
FSW	0.45 μm-filtered seawater
*H*_*C*_	Heterotrophic carbon grazing rate (μg C cm^−2^ h^−1^)
HTF	High temperature and fed corals
HTU	High temperature and unfed corals
*M*_*C*_	Mass of carbon per milligram of host tissue or symbionts (μg mg^−1^)
*M*_*sample*_	Mass of the freeze-dried sample (mg)
*M*_*Sk*_	Amount of CaCO_3_ produced by calcification (μg CaCO_3_ cm^−2^ h^−1^)
*P*_*C*_	Amount of autotrophic carbon fixed by gross photosynthesis (μg C cm^−2^ h^−1^)
*P*_*g*_	Oxygen produced by gross photosynthesis (μmol O_2_ cm^−2^ h^−1^)
*P*_*n*_	Oxygen produced by net photosynthesis (μmol O_2_ cm^−2^ h^−1^)
*PQ*	Photosynthetic quotient (equal to 1.1 mol O_2_:mol C)
*R*	Oxygen consumed by respiration of holobiont (μmol O_2_ cm^−2^ h^−1^)
*R*_*C*_	Amount of carbon respired by holobiont (μg C cm^−2^ h^−1^)
*R*_*H*_	Amount of carbon respired by coral host (μg C cm^−2^ h^−1^)
*RQ*	Respiratory quotient (equal to 0.8 mol C: mol O_2_)
*R*_*S*_	Amount of autotrophic carbon respired by symbionts (μg C cm^−2^ h^−1^)
S	Nubbin surface area (cm^2^)
*T*_*S*_	Autotrophic carbon translocated from symbionts to their host (μg C cm^−2^ h^−1^or %)
*t*_*chase*_	Incubation time of the nubbins in the non-enriched medium in the light (equal to 24 h)
*t*_*pulse*_	Incubation time of the nubbins in the enriched medium (equal to 5 h)
*ρ*_*DOC*_	Amount of autotrophic carbon lost in released dissolved organic carbon (μg C cm^−2^ h^−1^)
*ρ*_*H*_	Amount of autotrophic carbon incorporated in the coral host (μg C cm^−2^ h^−1^)
*ρ*_*POC*_	Amount of autotrophic carbon lost in released particulate organic carbon (μg C cm^−2^ h^−1^)
*ρ*_*S*_	Amount of autotrophic carbon incorporated in the symbionts (μg C cm^−2^ h^−1^)
