# Supplementary material for: Heterotrophy promotes the re-establishment of photosynthate translocation in a symbiotic coral after heat stress
Source: Sci Rep. 2016 Dec 5;6:38112. doi: 10.1038/srep38112 (PMC5137022; doi:10.1038/srep38112)
Supplement: Supplementary Information [file srep38112-s1.pdf]

Pascale Tremblay, Andrea Gori, Jean François Maguer, Mia Hoogenboom and Christine Ferrier-Pagès

**Table S1** Symbiont concentration, gross photosynthesis, $P_C$, holobiont respiration, $R_C$, host respiration, $R_H$, symbiont respiration, $R_S$, and calcification, $C_C$, for fed and unfed nubbins of *Stylophora pistillata* before the start of experiment and heat stress (day 0). Data are expressed as means ± standard error of the mean of $n$ = 4 measurements. CTF: control temperature at 25°C and fed; CTU: control temperature at 25°C and unfed; HTF: high temperature at 31°C and fed; and HTU: high temperature at 31°C and unfed.

| | Symbiont concentration ($10^6$ cells $cm^{-2}$) | | | Photosynthesis, $P_C$ (µg C $cm^{-2}$ $h^{-1}$) | | | Holobiont respiration, $R_C$ (µg C $cm^{-2}$ $h^{-1}$) | | | Host respiration, $R_H$ (µg C $cm^{-2}$ $h^{-1}$) | | | Symbiont respiration, $R_S$ (µg C $cm^{-2}$ $h^{-1}$) | | | Calcification, $C_C$ (µg C $cm^{-2}$ $h^{-1}$) | | |
|---|---|---|---|---|---|---|---|---|---|---|---|---|---|---|---|---|---|---|
| | mean | ± | s.e.m. | mean | ± | s.e.m. | mean | ± | s.e.m. | mean | ± | s.e.m. | mean | ± | s.e.m. | mean | ± | s.e.m. |
| Day 0 | | | | | | | | | | | | | | | | | | |
| CTF | 1.98 | ± | 0.18 | 24.1 | ± | 0.4 | 12.8 | ± | 0.7 | 10.9 | ± | 0.6 | 1.9 | ± | 0.1 | 14.2 | ± | 0.5 |
| CTU | 1.66 | ± | 0.20 | 17.8 | ± | 3.3 | 11.1 | ± | 1.3 | 9.9 | ± | 1.2 | 1.2 | ± | 0.1 | 10.9 | ± | 0.5 |
| HTF | 1.84 | ± | 0.17 | 21.9 | ± | 1.1 | 14.2 | ± | 0.9 | 12.1 | ± | 1.0 | 2.1 | ± | 0.2 | 13.7 | ± | 0.3 |
| HTU | 1.38 | ± | 0.10 | 17.7 | ± | 1.4 | 9.8 | ± | 0.4 | 8.5 | ± | 0.5 | 1.3 | ± | 0.1 | 12.2 | ± | 0.9 |

**Table S2** Percentages of autotrophic carbon contributing to the total respiration of the holobiont (CZAR) as well as percentages of autotrophic and heterotrophic carbon contributing to the respiration of the host (CTAR and CHAR respectively), for fed and unfed nubbins of *Stylophora pistillata* before the start of experiment and heat stress (day 0). Data are expressed as means ± standard error of the mean of *n* = 4 measurements. CTF: control temperature at 25°C and fed; CTU: control temperature at 25°C and unfed; HTF: high temperature at 31°C and fed; and HTU: high temperature at 31°C and unfed.

| | CZAR (%) | | | CTAR (%) | | | CHAR (%) | | | CTAR + CHAR (%) | | |
|---|---|---|---|---|---|---|---|---|---|---|---|---|
| | mean | ± | s.e.m.(Fisher) | mean | ± | s.e.m.(Fisher) | mean | ± | s.e.m.(Fisher) | mean | ± | s.e.m.(Fisher) |
| Day 0 | | | | | | | | | | | | |
| CTF | 94.8 | ± | 3.4 | 92.2 | ± | 5.2 | 25.7 | ± | 1.4 | 118.0 | ± | 6.6 |
| CTU | 79.6 | ± | 5.3 | 78.2 | ± | 9.3 | | | | 78.2 | ± | 9.3 |
| HTF | 77.9 | ± | 6.6 | 76.4 | ± | 6.0 | 23.3 | ± | 1.8 | 99.7 | ± | 7.9 |
| HTU | 90.4 | ± | 8.4 | 89.9 | ± | 5.0 | | | | 89.9 | ± | 5.0 |
